# *SpEDIT*: A fast and efficient CRISPR/Cas9 method for fission yeast

**DOI:** 10.12688/wellcomeopenres.16405.1

**Published:** 2020-11-24

**Authors:** Sito Torres-Garcia, Lorenza Di Pompeo, Luke Eivers, Baptiste Gaborieau, Sharon A. White, Alison L. Pidoux, Paulina Kanigowska, Imtiyaz Yaseen, Yizhi Cai, Robin C. Allshire

**Affiliations:** 1 Wellcome Centre for Cell Biology and Institute of Cell Biology, School of Biological Sciences, University of Edinburgh, Mayfield Road, Edinburgh, EH9 3BF, UK; 2 School of Biological Sciences, University of Edinburgh, Mayfield Road, Edinburgh, EH9 3BF, UK; 3 Present address: Manchester Institute of Biotechnology, University of Manchester, Manchester, M1 7DN, UK

**Keywords:** CRISPR/Cas9, gene editing, gene tagging, S. pombe, fission yeast

## Abstract

The CRISPR/Cas9 system allows scarless, marker-free genome editing. Current CRISPR/Cas9 systems for the fission yeast *Schizosaccharomyces pombe *rely on tedious and time-consuming cloning procedures to introduce a specific sgRNA target sequence into a Cas9-expressing plasmid. In addition, Cas9 endonuclease has been reported to be toxic to fission yeast when constitutively overexpressed from the strong *adh1 *promoter. To overcome these problems we have developed an improved system, *SpEDIT*, that uses a synthesised Cas9 sequence codon-optimised for *S. pombe *expressed from the medium strength *adh15 *promoter. The *SpEDIT* system exhibits a flexible modular design where the sgRNA is fused to the 3’ end of the self-cleaving hepatitis delta virus (HDV) ribozyme, allowing expression of the sgRNA cassette to be driven by RNA polymerase III from a tRNA gene sequence. Lastly, the inclusion of sites for the *Bsa*I type IIS restriction enzyme flanking a GFP placeholder enables one-step Golden Gate mediated replacement of GFP with synthesized sgRNAs for expression. The *SpEDIT* system allowed a 100% mutagenesis efficiency to be achieved when generating targeted point mutants in the *ade6 ^+^* or *ura4* ^+^ genes by transformation of cells from asynchronous cultures. *SpEDIT* also permitted insertion, tagging and deletion events to be obtained with minimal effort. Simultaneous editing of two independent non-homologous loci was also readily achieved. Importantly the *SpEDIT* system displayed reduced toxicity compared to currently available *S. pombe* editing systems. Thus, *SpEDIT *provides an effective and user-friendly CRISPR/Cas9 procedure that significantly improves the genome editing toolbox for fission yeast.

## Introduction

The fission yeast *Schizosaccharomyces pombe* is a powerful model organism widely used in cellular and molecular biology ( Fantes & Hoffman, 2016). Traditionally, gene manipulation in *S. pombe* is achieved by transforming a DNA construct that includes the desired change alongside a selectable marker. The DNA construct integrates in the genome via flanking regions that target the genomic locus of interest. DNA constructs vary depending on the application but commonly consist of a sole PCR product that comprises an insertion, deletion or tagging cassette amplified from an existing plasmid ( Bähler *et al.*, 1998). Albeit convenient, this approach results in a selectable marker integrated at the target locus, consequently disrupting the local genomic context and limiting the availability of markers for subsequent manipulations.

The prokaryotic CRISPR/Cas9 system enables flexible and scarless genome editing without the necessity of selectable markers escorting the introduced DNA change and disturbing the local genomic environment ( Hsu *et al.*, 2014; Jinek *et al.*, 2012). Adapted from a genome defence mechanism against invading DNA, the engineered minimal CRISPR/Cas9 system consists of a Cas9 endonuclease and a single-guide RNA (sgRNA) chimera that contains both the trans-activating CRISPR RNA and the targeting CRISPR RNA ( Jinek *et al.*, 2012). The sgRNA sequence targets the system to a defined genomic location where the Cas9 endonuclease binds to a protospacer adjacent motif (PAM). The Cas9 enzyme then creates a double-strand break (DSB) three base pairs upstream of the PAM site in the protospacer sequence ( Jinek *et al.*, 2012). Following DSB generation, repair is executed either through error-prone non-homologous end-joining (NHEJ), where strand end resection and subsequent repair frequently induce indels, or through high-fidelity homology-directed repair (HDR). HDR involves recombination via sequence homology and therefore can be exploited to generate precise mutations by providing a DNA editing template that contains the required DNA change and engages in homologous recombination (HR) with the cleaved region ( Hsu *et al.*, 2014).

Implementation of the CRISPR/Cas9 system in *S. pombe* has been previously described in several reports ( Fernandez & Berro, 2016; Hayashi & Tanaka, 2019; Jacobs *et al.*, 2014; Rodríguez-López *et al.*, 2016; Zhao & Boeke, 2018; Zhang *et al.*, 2018). A useful web-tool, CRISPR4P, was also developed to support the design of sgRNAs and oligonucleotides required to perform CRISPR/Cas9-mediated gene deletions ( Rodríguez-López *et al.*, 2016).

To date, all CRISPR/Cas9 systems for *S. pombe* utilise the promoter/leader sequence of K RNA ( *rrk1*) and a hammerhead ribozyme (HHR) for sgRNA cassette expression ( Jacobs *et al.*, 2014). A humanised Cas9 endonuclease expressed from the strong *adh1* promoter was used in the original *S. pombe* system ( Jacobs *et al.*, 2014). The resulting high levels of the Cas9 endonuclease were found to be detrimental for *S. pombe* growth. This Cas9 toxicity was partially bypassed by co-expression of the sgRNA and the Cas9 enzyme from a single plasmid ( Jacobs *et al.*, 2014).

Cloning of a sgRNA target into the single sgRNA/Cas9 plasmid was originally executed via *Csp*CI digestion ( Jacobs *et al.*, 2014). This procedure proved to be extremely inefficient due to the large plasmid size and the inconsistency in available commercial *Csp*CI preparations ( Rodríguez-López *et al.*, 2016). A subsequent study attempted to overcome this problem by implementing a PCR-based method in which a sgRNA target is introduced into a single sgRNA/Cas9 plasmid by using overlapping PCR primers that contain the sgRNA sequence (pMZ379 plasmid, Rodríguez-López *et al.*, 2016). Although an improvement over the initial system, this PCR-based method generated only a low frequency of bacterial colonies that contain correct and intact constructs. As a consequence, the required screening makes the entire process inefficient and time consuming.

To circumvent issues pertaining to Cas9 toxicity and inefficient sgRNA cloning procedures, here we report the development of *SpEDIT*, an improved CRISPR/Cas9 system for the efficient manipulation of the fission yeast genome. *SpEDIT* employs a highly effective one-step Golden Gate cloning strategy for the insertion of sgRNAs, that in combination with the use of a GFP placeholder allows visual screening for identification of positive clones. The Cas9 endonuclease gene implemented in this system is codon-optimised for expression in *S. pombe* and driven by the medium strength promoter *adh15*, resulting in reduced toxicity associated with Cas9 levels. *SpEDIT* can generate targeted *ade6* and *ura4* point mutants in asynchronous cells with 100% mutagenesis efficiency. Moreover, *SpEDIT* allows simultaneous editing at two non-homologous genes at distinct locations in the *S. pombe* genome, as well as seamless insertion, deletion and tagging at *S. pombe* loci. *SpEDIT* provides an efficient and simple CRISPR/Cas9 method to easily manipulate the genome of the fission yeast.

## Results

### The *SpEDIT* system

**Figure 1.**
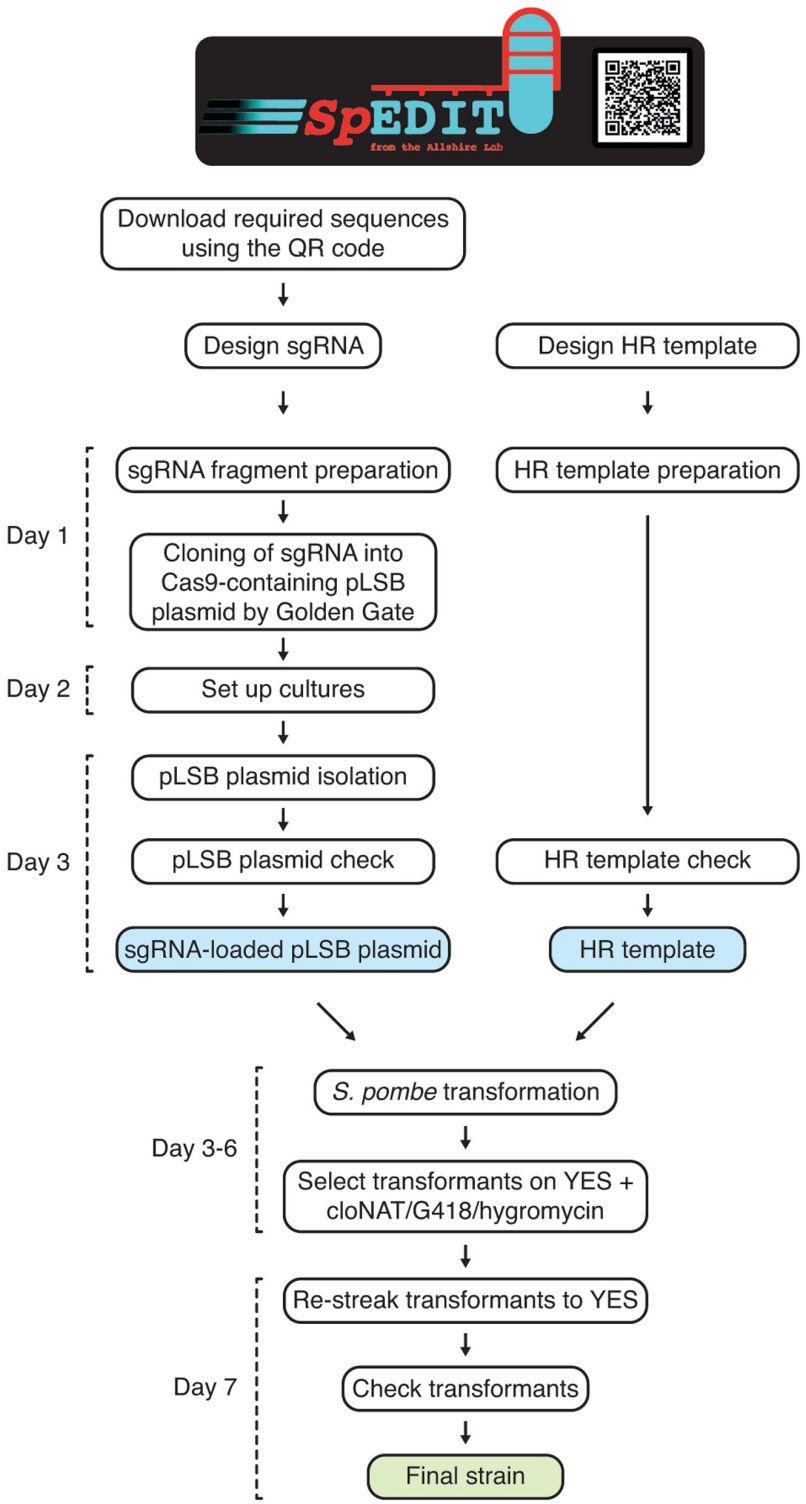
*SpEDIT* provides a fast and effective CRISPR/Cas9 method to manipulate the genome of *Schizosaccharomyces pombe.* Diagram illustrating the required steps for *S. pombe* strain construction using *SpEDIT*. For a full protocol see methods. sgRNA, single guide RNA. HR template, homologous recombination donor template.

The *SpEDIT* system has been developed to address the two main complications associated with existing CRISPR/Cas9 methods for *S. pombe*: toxicity associated with Cas9 overexpression, and laborious cloning procedures required to insert a specific sgRNA target sequence into a Cas9-containing plasmid. An overview of the *SpEDIT* system is provided ( Figure 1) along with a full protocol (see Methods).

High levels of human codon-optimised Cas9 endonuclease constitutively expressed from the exceptionally strong *adh1* promoter (400 RNA molecules/cell, PomBase, Lock *et al.*, 2019) lead to reduced cell growth in *S. pombe* ( Jacobs *et al.*, 2014). A recent report attempted to solve this toxicity problem by expressing the human codon-optimised Cas9 under control of the repressible *nmt41* promoter ( Hayashi & Tanaka, 2019). Although this approach does generate mutations, it requires the non-ideal use of minimal media and relies on auxotrophic ( *ura4 ^-^* or *leu1 ^-^*) strains to allow plasmid selection. Moreover, the mutagenesis efficiency obtained was dependent on the selectable marker employed.

**Figure 2.**
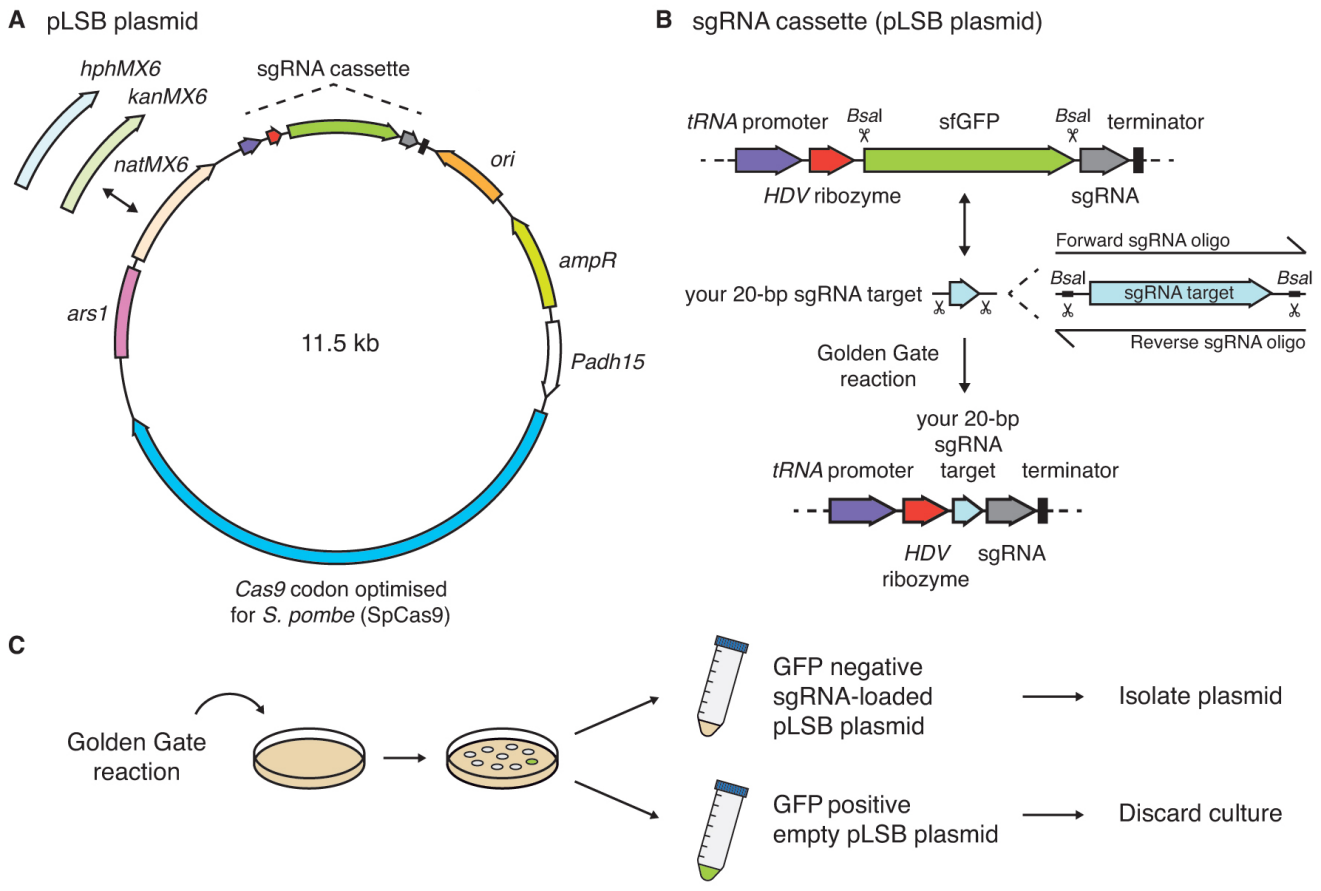
The *SpEDIT* pLSB plasmid allows one-step insertion of sgRNAs via Golden Gate cloning. **A**. Map of pLSB plasmid **.**Full sequence is available scanning the QR code in Figure 1 or at *allshirelab.com/spedit*. Versions with *natMX6* (cloNAT), *kanMX6* (G418) or *hphMX6* (hygromycin) *S. pombe* resistance markers are available. A Cas9 codon optimised for *S. pombe* (SpCas9) is expressed from the *adh15* promoter ( *Padh15*). **B**. Diagram of sgRNA cassette and cloning procedure. sgRNA cassette expression is driven by a tRNA ^Ser^ Pol III promoter (purple block arrow). A self-cleaving hepatitis delta virus (HDV) ribozyme is located at the 5’ end of the sgRNA cassette ( Ryan *et al.*, 2014) (red block arrow). A superfolder green fluorescent protein (sfGFP) is used as placeholder (green block arrow). *Bsa*I sites flanking sfGFP allow one-step insertion of a sgRNA target (light blue block arrow) into the sgRNA scaffold (grey block arrow) via Golden Gate cloning. The Pol III terminator sequence from *S. cerevisiae SUP4* (tRNA ^Tyr^) is present at the 3’ end of the sgRNA cassette (black block). **C**. The sfGFP placeholder allows cultures carrying empty (green) pLSB plasmids to be distinguished from sgRNA-loaded (non-green) pLSB plasmids. sgRNA, single guide RNA.

To overcome the toxicity related to high levels of humanised Cas9, we synthesized a Cas9 gene codon-optimised for expression in *S. pombe* (SpCas9) that is transcribed from the medium strength *adh15* promoter ( Yamagishi *et al.*, 2008). This *adh15-*SpCas9 gene is carried on a new plasmid, pLSB, that contains a choice of dominant selectable markers. Versions bearing *natMX6, kanMX6* or *hphMX6* markers are available, thereby allowing the *SpEDIT* system to be employed on fission yeast strains that harbour various manipulations where other selectable markers are already present ( Figure 2A).

Eukaryotic CRISPR/Cas9 systems usually rely on snRNA or snoRNA RNA polymerase III (RNAPIII) promoters for transcription of the sgRNA cassette ( Cong *et al.,* 2013). However, characterised *S. pombe* RNAPIII genes contain promoter elements within the transcribed region, preventing their use for generating accurately positioned 5’ ends, and RNA polymerase II (RNAPII) promoters generally do not generate transcripts with precise 5’ and 3’ ends. Consequently, all sgRNA expression systems for *S. pombe* so far utilise the *rrk1* promoter plus its downstream 5’ untranslated region which generates a RNAPII transcript with a cleavable leader RNA ( Jacobs *et al.*, 2014). Insertion of sgRNA sequences targeting genomic regions of interest into the *rrk1* sgRNA expression cassette in current CRISPR/Cas9 systems for *S. pombe* relies on slow and arduous cloning procedures involving either traditional restriction digestion ( Hayashi & Tanaka, 2019; Jacobs *et al.*, 2014) or PCR over a long template ( Rodríguez-López *et al.*, 2016). An alternative method that uses *in vivo* gap repair to assemble a gapped Cas9-encoding plasmid and a PCR-amplified sgRNA fragment into a single circular plasmid has been reported ( Zhang *et al.*, 2018). Although this method provided an advance, this system still utilises humanised Cas9 expressed from the very strong *adh1* promoter, which consequently reduces cell growth due to Cas9-associated toxicity.

It has previously been shown that the upstream tRNA ^Ser^ gene of an *S. pombe* tRNA ^Ser^-tRNA ^Met^ gene pair drives RNAPIII expression of the downstream tRNA ^Met^ gene ( Hottinger-Werlen *et al.*, 1985). We therefore use this tDNA ^Ser^ to drive sgRNA expression in *S. pombe*. The resulting *SpEDIT* system employs a modular design where sgRNAs are expressed from this tDNA ^Ser^ sequence and fused to the hepatitis delta virus ribozyme (HDV), as previously described for *Saccharomyces cerevisiae* ( Ryan *et al.*, 2014). The tDNA ^Ser^ acts as a RNAPIII promoter and the self-cleaving ribozyme protects and defines the 5’ end of the resulting sgRNA ( Figure 2A and B). The presence of the HDV ribozyme was shown to result in a six-fold increase in sgRNA abundance and this correlated with high targeting efficiency in *S. cerevisiae* ( Ryan *et al.*, 2014). To facilitate cloning of sgRNA target sequences into this tDNA/HDV expression cassette, we placed sites for the *Bsa*I type IIS restriction enzyme on each side of a GFP placeholder, thereby allowing one-step insertion of sgRNAs via Golden Gate cloning. Importantly, this strategy also permits visual screening to identify colonies that have lost the green GFP fluorescence signal indicating that the GFP has been successfully replaced with an incoming sgRNA ( Figure 2B and C).

### *SpEDIT* can generate targeted *ade6* and *ura4* point mutants in asynchronous cells with 100% mutagenesis efficiency

**Figure 3.**
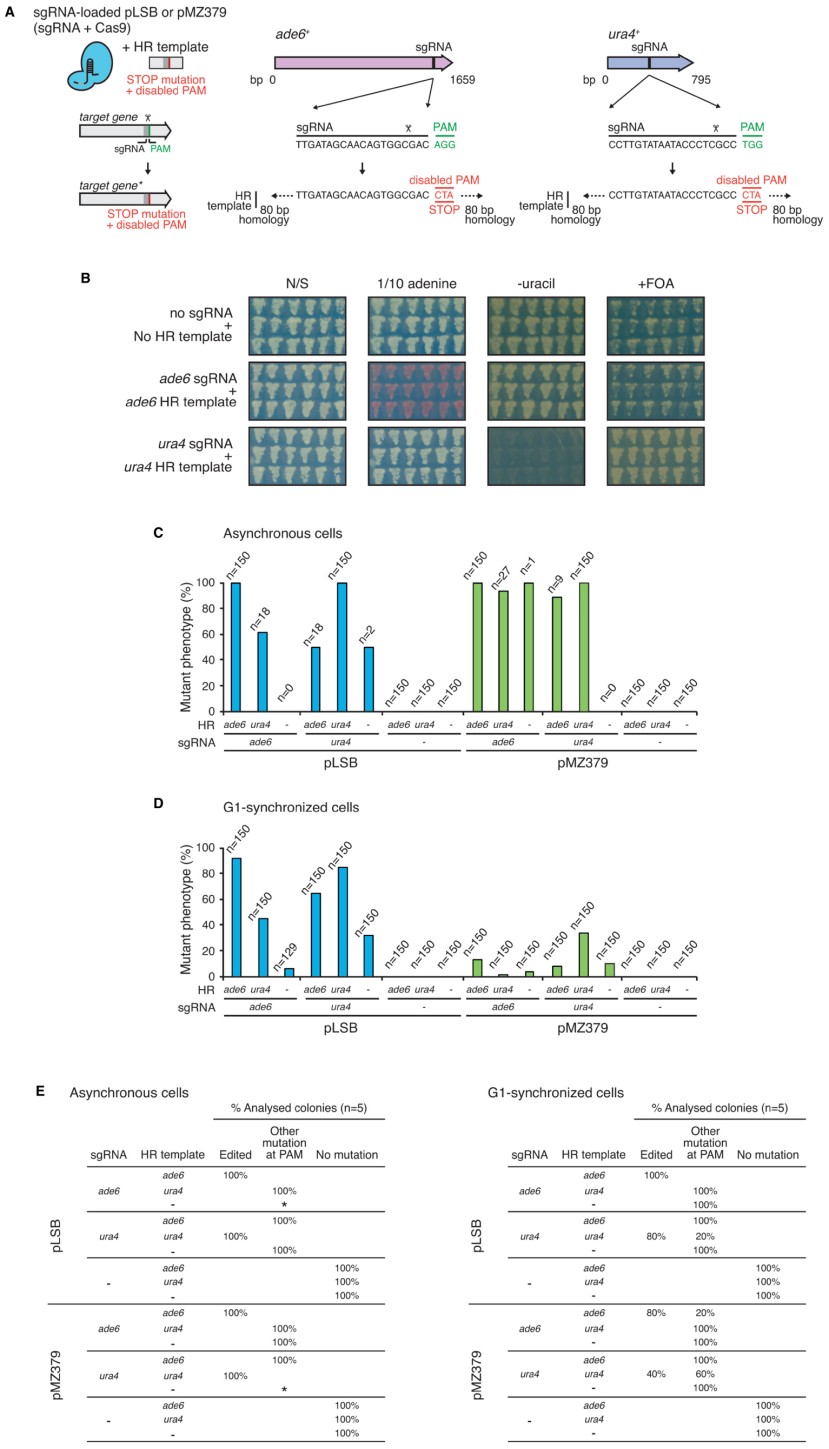
*SpEDIT* can generate targeted *ade6* and *ura4* point mutants in asynchronous cells with 100% mutagenesis efficiency. **A**. Schematic of experiment to generate targeted *ade6* and *ura4* point mutants. A sgRNA-loaded pLSB or pMZ379 plasmid was co-transformed with an HR template that creates a premature STOP codon by disabling the PAM (NGG) sequence. sgRNA and HR template sequences for *ade6* and *ura4* are shown. Full HR template sequences can be found in Table 1. **B**. After transformation, cloNAT-resistant colonies were picked and re-streaked to non-selective YES plates. Cells were then replica-plated to indicated media to assess their phenotype. Representative plates from two independent experiments are shown. Quantification is shown in **C**– **D**. **C**– **D**. Percentage of cloNAT-resistant transformants displaying a mutant phenotype (pink cells, *ade6;* uracil auxotrophy and FOA resistance, *ura4*) after asynchronous ( **C**) or G1-synchronized ( **D**) wild-type cells were transformed with a sgRNA-loaded *SpEDIT/*pLSB (developed here, Figure 2) or pMZ379 ( Rodríguez-López *et al.*, 2016) plasmid targeting *ade6 ^+^* or *ura4 ^+^* (or no sgRNA plasmid as control). An HR template targeting the same or a different gene was co-transformed as indicated. n = number of cloNAT-resistant colonies assayed. Note that when an HR template targeting a different gene or no HR template was co-transformed into asynchronous cells, the number of cloNAT-resistant colonies obtained was drastically reduced. Experiment was repeated twice with similar results. **E**. For each condition in **C**– **D**, five colonies displaying the mutant phenotype (or 5 cloNAT-resistant colonies for no sgRNA plasmids) were taken and the gene targeted by the sgRNA was sequenced to confirm changes in its DNA sequence. Both *ade6* and *ura4* were sequenced when no sgRNA was used. Edited clones harbour the change contained in the corresponding HR template. Other mutations at PAM disrupt the PAM (NGG) sequence and the corresponding gene coding sequence. For asynchronous cells transformed with pLSB- *ura4* (no HR template) and pMZ379- *ade6* (no HR template) only two and one colonies were respectively obtained and analysed. * No colonies were obtained for these conditions. sgRNA, single guide RNA. PAM, proto-spacer adjacent motif. HR, homologous recombination donor template. N/S, non-selective medium. FOA, 5-fluoroorotic acid.

To assess the performance of the *SpEDIT* system in comparison to the existing pMZ379 system ( Rodríguez-López *et al.*, 2016), we targeted the *ade6 ^+^* and *ura4 ^+^* genes and provided HR templates that disable the PAM (NGG) sequence downstream of the sgRNA target to generate premature STOP codon mutations ( Figure 3A). *ade6 ^+^* and *ura4 ^+^* mutations can be easily scored due to their characteristic phenotypes: *ade6* mutants, pink colonies develop on low (1/10) adenine-containing plates; *ura4* mutants, cannot grow in the absence of supplementing uracil (uracil auxotrophy) but can grow in the presence of counterselective 5-fluoroorotic acid (FOA resistant) ( Figure 3B).

We scored cloNAT-resistant colonies after electroporation of asynchronous cultures with either *SpEDIT*/pLSB or pMZ379 plasmids expressing sgRNA designed to mediate cleavage within the *ade6 ^+^* or *ura4 ^+^* genes in the presence or absence of an HR template homologous to *ade6 ^+^* or *ura4 ^+^*, respectively. The results revealed that both pLSB and pMZ379 plasmids can generate targeted mutations in *ade6 ^+^* and *ura4 ^+^* with 100% efficiency when a matching HR template was co-transformed ( Figure 3C). However, when an HR template targeting a different gene or no HR template was provided, the number of cloNAT-resistant colonies obtained was dramatically reduced. This decrease in transformant number in the absence of an HR template is consistent with futile cleavage-repair cycles where the persistence of a double strand break prevents cell division and thus colony formation ( Figure 3C).

Sequence analysis of the resulting *ade6* and *ura4* mutants showed that when a matching HR template was co-transformed, all clones analysed harboured the mutation provided by the HR template ( Figure 3E, left). However, a variety of mutations that disable the PAM sequence and ultimately disrupt the coding sequence of each gene were detected in mutants generated when a non-homologous HR template (targeting a different gene to the sgRNA) or no HR template was provided ( Figure 3E, left).

A previous study suggested that G1 synchronization of *S. pombe* cultures by nitrogen starvation prior to CRISPR/Cas9-mediated genome editing enhances transformation and deletion efficiencies ( Rodríguez-López *et al.*, 2016). The rationale for this was that in G1 only one copy of a target locus would need to be modified as opposed to the two copies that are present in G2 cells. The remodelled transcriptional programme of G1 cells was also expected to render genomic regions more open ( Mata *et al.*, 2002), and thus increase accessibility to the editing machinery.

We therefore compared the performance of the *SpEDIT* system (pLSB plasmid) versus the existing pMZ379 system (pMZ379 plasmid) at generating *ade6* and *ura4* mutations using G1-synchronized *S. pombe* cells as previously described ( Rodríguez-López *et al.*, 2016). This comparison revealed that the pLSB plasmid is more efficient than the pMZ379 plasmid at generating mutations in G1-synchronized cells ( Figure 3D). However, even when a matching HR template was co-transformed, the mutagenesis efficiencies obtained with G1 cells were lower than that of asynchronous cells (pLSB→ ade6, 92% G1 versus 100% Asynchronous; pLSB→ ura4, 85% G1 versus 100% Asynchronous) ( Figure 3D).

Moreover, sequence analysis revealed that even when a matching HR template was co-transformed into G1 cells, not all mutant clones harboured the anticipated mutation that was presented by the HR template ( Figure 3E, right). This lack of accuracy is likely due to the suppression of the HR pathway that is known to occur in G1 cells ( Orthwein *et al.*, 2015; Trickey *et al.*, 2008).

Taken together, our results show that the *SpEDIT* system can generate targeted mutations at *ade6 ^+^* and *ura4 ^+^* with 100% mutagenesis efficiency using asynchronous cell cultures. Notably, our experiments show that G1 synchronization of *S. pombe* cells prior transformation has a detrimental effect on mutagenesis efficiency regardless of the CRISPR/Cas9 system used.

### *SpEDIT* shows reduced toxicity compared with the current pMZ379 CRISPR/Cas9 system in asynchronous cells

**Figure 4.**
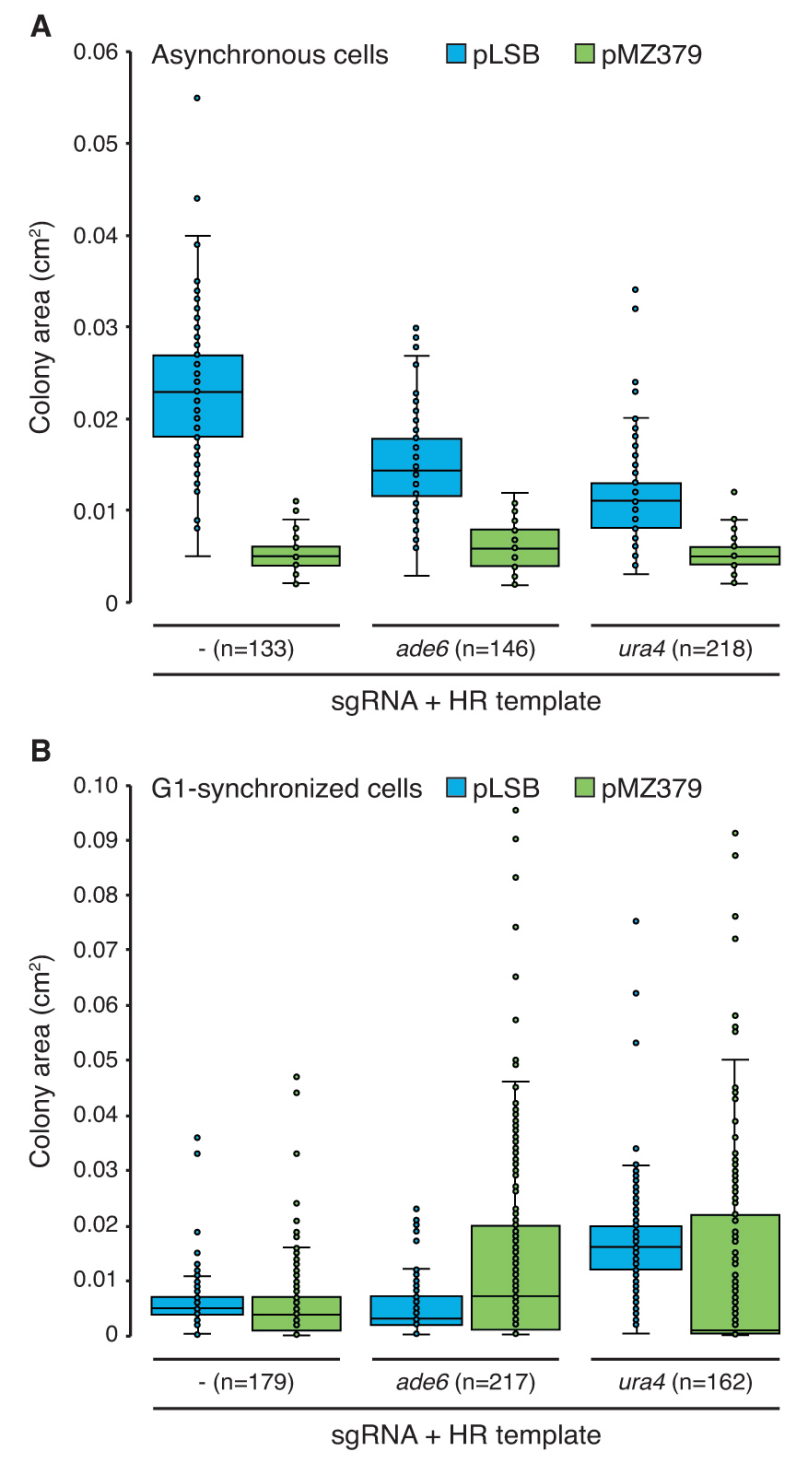
*SpEDIT* shows reduced toxicity compared with the current pMZ379 *S. pombe* CRISPR/Cas9 system in asynchronous cells. **A**– **B**. Colony area measurements of asynchronous ( **A**) or G1-synchronized ( **B**) wild-type cells transformed with pLSB or pMZ379 plasmids and indicated HR templates growing on selective cloNAT-containing plates (same experiment as Figure 3). Colony area was quantified (cm ^2^) using ImageJ. sgRNA, single guide RNA. HR, homologous recombination donor template.

To determine if the *SpEDIT* system leads to reduced toxicity compared to the current pMZ379 system, we measured colony area on selection plates after transforming asynchronous or G1-synchronized cultures with pLSB or pMZ379 plasmids targeting *ade6 ^+^* or *ura4 ^+^* (or empty plasmid controls) in the presence of a matching HR template. The colony area (equivalent to colony size) was found to be greater when asynchronous cells were transformed with pLSB as opposed to pMZ379 ( Figure 4A). The resulting difference in colony size was independent of the presence of a sgRNA, indicating that excessive levels of Cas9 alone, and not Cas9 targeting to a genomic locus, are sufficient to cause the observed toxicity ( Figure 4A). Consistent with this, the toxicity of *adh1-*Cas9 has also been shown to be independent of Cas9 catalytic activity ( Ciccaglione, 2015).

In contrast, colony area measurements of pLSB and pMZ379 transformants obtained from G1-synchronized cells revealed no major difference in resulting colony size ( Figure 4B). This indicates that the toxicity related to high levels of catalytically active Cas9 is more prominent when transforming asynchronous cells. The lack of apparent toxicity in G1 cells is likely due to the known upregulation of non-homologous end joining-mediated repair and the suppression of homologous recombination repair that is known to occur at this cell cycle stage ( Orthwein *et al.*, 2015).

### *SpEDIT* allows simultaneous editing at two non-homologous genes at distinct locations in the *S. pombe* genome

The availability of pLSB versions bearing different dominant selectable markers presented the opportunity to test if the simultaneous editing of two different non-homologous loci by co-transformation with and selection of two distinct pLSB plasmids expressing different sgRNAs was possible.

**Figure 5.**
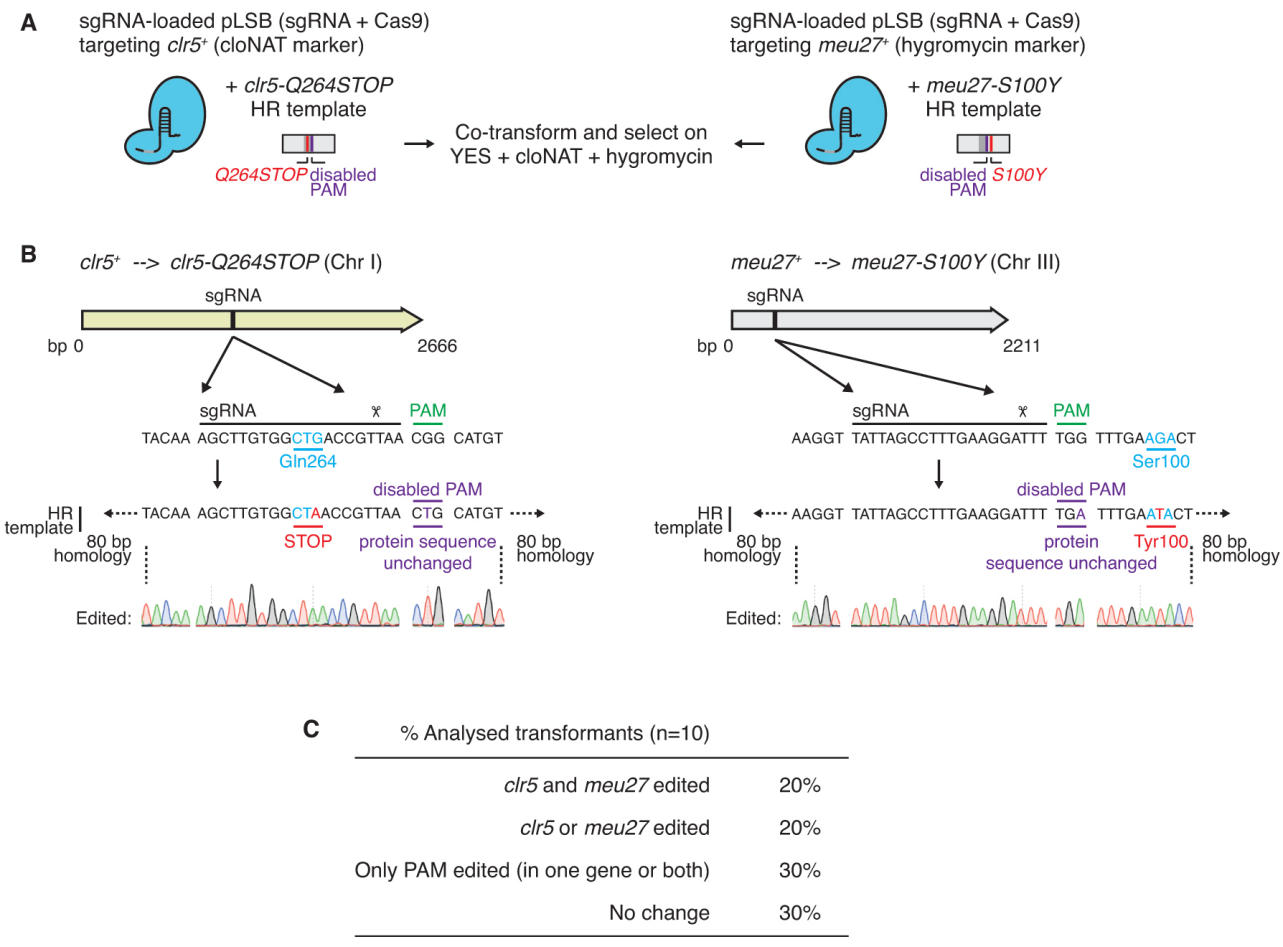
*SpEDIT* allows simultaneous editing at two non-homologous genes at distinct locations in the *S. pombe* genome. **A**. Schematic of experiment to simultaneously generate targeted point mutations in *clr5 ^+^* and *meu27 ^+^*. Two sgRNA-loaded pLSB plasmids (with different selection markers) were co-transformed with two HR templates that create the desired point mutations and disable the corresponding PAM (NGG) sequence. Transformed cells were then selected on selective plates containing both cloNAT and hygromycin. **B**. sgRNA and HR template sequences for *clr5* (left) and *meu27* (right) are shown, along with Sanger sequencing chromatograms for a successfully edited clone. Full HR template sequences can be found in Table 1. **C**. Percentage of cloNAT- and hygromycin-resistant transformants harbouring the targeted mutations in *clr5* and *meu27* as revealed by Sanger sequencing. sgRNA, single guide RNA. HR, homologous recombination donor template.

To evaluate the possibility of simultaneous editing, we targeted two non-homologous genes, *clr5 ^+^* and *meu27 ^+^*, located on different chromosomes (I and III, respectively) in wild-type cells using pLSB-cloNAT and pLSB-hygromycin plasmids that express sgRNAs designed to target *clr5 ^+^* and *meu27 ^+^*, respectively ( Figure 5A). We co-transformed two HR templates that generate point mutations in *clr5 ^+^* ( *clr5-Q264STOP*) and *meu27 ^+^* ( *meu27-S100Y*), and concomitantly disabled both corresponding PAM sequences ( Figure 5A and B). These mutations ( *clr5-Q264STOP* and *meu27-S100Y*) had previously been identified in a proportion of heterochromatin-dependent epimutants resistant to caffeine ( Torres-Garcia *et al.,* 2020).

Sequencing of *clr5* and *meu27* in ten resulting cloNAT and hygromycin doubly resistant co-transformants revealed that two harboured both of the expected DNA changes. Five clones carried mutations in only one of the two targeted genes or bore mutations that uniquely affected the PAM sequence, and three clones displayed neither of the anticipated changes ( Figure 5C). Importantly, whole-genome sequencing of one of the isolates that contained both expected gene editing events revealed no additional genetic changes (SNPs or indels) in coding regions of the genome ( Torres-Garcia *et al.,* 2020).

These results demonstrate that our improved system can be utilised to perform simultaneous gene editing at two distant, non-homologous *S. pombe* loci, albeit with reduced efficiency relative to the frequency of editing of a single locus.

### Seamless insertion, deletion and tagging at *S. pombe* loci using *SpEDIT*

**Figure 6.**
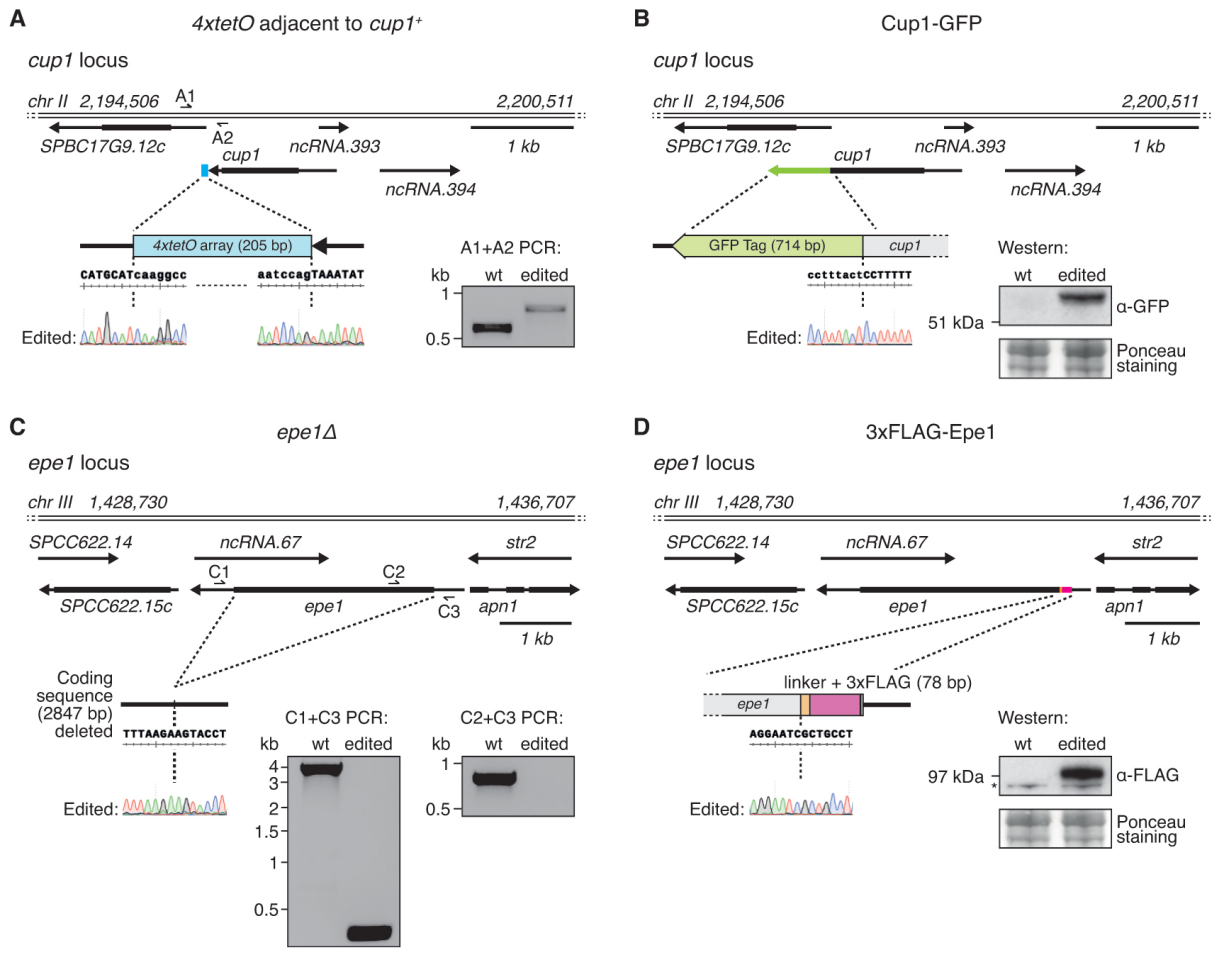
*SpEDIT* allows seamless insertion, deletion and tagging at *S. pombe* loci. For sgRNA and HR template sequences see Table 1. **A**. *4xtetO* binding sites were inserted downstream of *cup1 ^+^*. Sanger sequencing chromatograms covering the insert junctions are shown for a successfully edited clone. PCR primers (half arrows) flanking the insert were used to amplify products from wild-type (wt) and edited strains. **B**. Cup1 was C-terminally tagged with a green fluorescent protein (GFP). Sanger sequencing chromatogram covering the gene-tag junction is shown for a successfully edited clone. Western blot using anti-GFP antibody was performed on wild-type (wt) and edited strains. **C**. The coding sequence of *epe1 ^+^* was deleted. Sanger sequencing chromatogram covering the deletion junction is shown for a successfully edited clone. PCR primers (half arrows) flanking the deletion (and within the *epe1 ^+^* coding sequence as control) were used to amplify products from wild-type (wt) and edited strains. **D**. Epe1 was N-terminally tagged with three FLAG epitopes. Sanger sequencing chromatogram covering the gene-tag junction is shown for a successfully edited clone. Western blot using anti-FLAG antibody was performed on wild-type (wt) and edited strains.

To assess the capabilities of *SpEDIT* in additional gene editing tasks, we utilised it to perform insertion, deletion and tagging at single *S. pombe* loci. Specifically, using *SpEDIT*, we inserted *tetO* binding sites downstream of the *cup1 ^+^* (SPBC17G9.13c) gene ( Figure 6A). *tetO* binding sites allow tethering of proteins such as TetR-Clr4* and heterochromatin formation in the vicinity of the tethering site ( Audergon *et al.*, 2015; Ragunathan *et al.*, 2015). A fusion-PCR construct containing *4xtetO* sites with 120 bp homology arms flanking the desired insertion site was used as the HR template (see Table 1 for sgRNA and HR template sequences). Correct insertion of the *cup1:4xtetO* HR template resulted in ablation of the PAM sequence. Furthermore, we used *SpEDIT* to seamlessly fuse GFP in frame with the 3’ end of the of *cup1* ^+^ gene to produce Cup1-GFP ( Figure 6B). To generate the *cup1-gfp* HR template, the *GFP* open reading frame was amplified with oligonucleotides that had long extensions homologous to sequence immediately up-stream and down-stream of the normal *cup1* ^+^ STOP codon. This HR template also carried a DNA change (in the 5’ long oligo) designed to disable the PAM sequence without altering the Cup1 protein sequence. Resulting strains were confirmed to carry the planned *cup1:4xtetO* or Cup1-GFP insertions in the absence of any associated selectable marker and have been utilised to show that heterochromatin-mediated silencing of *cup1 ^+^* is sufficient to drive caffeine resistance in wild-type cells and that Cup1-GFP localises to mitochondria ( Torres-Garcia *et al.*, 2020).

**Table 1.** sgRNA and HR templates used in this study. All sgRNA sequences used were obtained using CRISPR4P ( Rodríguez-López *et al.*, 2016).

Name	Sequence
*ade6* - sgRNA	TTGATAGCAACAGTGGCGAC
*ura4* - sgRNA	CCTTGTATAATACCCTCGCC
*meu27* - sgRNA	TATTAGCCTTTGAAGGATTT
*clr5* - sgRNA	AGCTTGTGGCTGACCGTTAA
*cup1:4xtetO* - sgRNA	ATTTCTTTTGCTTTACGGTC
cup1-GFP - sgRNA	GCTCAGGCTAAACGTCGGAA
*epe1* - sgRNA	GGACTTTTAAGATGGATTCC
*ade6* - HR template	GCAATGACACCTCTTCCAGTAATCGGCGTTCCTGTAAAAGGAAGCACTCTTGACGGAGTTGACTCTCTTTAGTCTATTGTTCA GATGCCTCGAGGTGTCTAGGTCGCCACTGTTGCTATCAATAATAGCCAAAATGCCGGTATTTTAGCCTGTCGTATACTTGCTACA TTTCAACCCTCC
*ura4* - HR template	TTGGAAGACATTTCAGCCAAAAGCAAGAGACCACGTCCCAAAGGTAAACCAACTTCTTTGAGGCCTTGTATAATACCCTCGCCC TACACTGTATGGCAATTTGTGATATGAGCCCAAGACTAAATTTTGTACACACCAGATGCATATTGTAGCTTGACGGTATTTCCAAT GTCTGCGAAT
*meu27* - HR template	GCCAAAATCAATAGAGAACAATTATACTTTAAAAAAAAAAAATGAAGAAGGCTTCTTAAGTCAACAGGAAAATAAGTATTCAAATC AAAATCCTTCAAAGGCTAATAACCTTTTCGACAAGCTCGGCGTAAGCAAGCCAATAACATTCATAGCTTGTATACTGCAATTGGGC GGCACCTC
*clr5* - HR template	CTTATTTGCAGCAGCCTTTCCAAATACCCTCTCAACGTTTCTCTCGACAGCAACAATCTCATCCATTCCCTGCTGCTCAACATGCA GTTAACGGTTAGCCACAAGCTTTGTATCCTTTCATCTACCAATCTAGAAATGTCCCAATGGGCTCCACCATGTTTGCTTCTTCAAA CCAATCTG
*cup1:4xtetO* - HR template	TTGAATTAATTCATAGAGTATGATAAAAATTGATAGTAAATTCATTGGTATACTAAAGTGATGTAGAAAATTAAGAAATCACATAGAC TACTTGAGTTACGATGATTTATTAGCATGCATcaaggcctactagtgcatgcatcgatagatcctctatcactgatagggagatctccctatcagtgata gagaggatctccctatcagtgatagagaggatctccctatcagtgatagagaggatctccctatcagtgatagagaggatcctctctatcactgataggg agatcttcaacttggtggtgaggtaaacgaaatccagTAAATATTTATGAAAAAAAAAATAAATGATTCATAACAAGCAGATGAAAATGATGA CGAATTAGGACTCTTCAAAATAAATGAAGATTATACATTACAAA
cup1-GFP - HR template	ATGACGAATTAGGACTCTTCAAAATAAATGAAGATTATACATTACAAACTTTGGTCTGACTTTTTAAAGCACACGATTTGctatttgta tagttcatccatgccatgtgtaatcccagcagctgttacaaactcaagaaggaccatgtggtctctcttttcgttgggatctttcgaaagggcagattgt gtggacaggtaatggttgtctggtaaaaggacagggccatcgccaattggagtattttgttgataatggtctgctagttgaacgcttccatcttcaatgt tgtgtctaattttgaagttaactttgattccattcttttgtttgtctgccatgatgtatacattgtgtgagttatagttgtattccaatttgtgtccaag aatgtttccatcttctttaaaatcaataccttttaactcgattctattaacaagggtatcaccttcaaacttgacttcagcacgtgtcttgtagttcccg tcatctttgaaaaatatagttctttcctgtacataaccttcgggcatggcactcttgaaaaagtcatgccgtttcatatgatctgggtatcttgaaaagc attgaacaccataagtgaaagtagtgacaagtgttggccatggaacaggtagttttccagtagtgcaaataaatttaagggtaagttttccgtatgttgc atcaccttcaccctctccactgacagaaaatttgtgcccattaacatcaccatctaattcaacaagaattgggacaactccagtgaaaagttcttctcct ttactCCTTTTTTTGACTGACTTTTTAAGAACCTTTCCGACGTTTAGCCTGAGCTGAATGTCTGACAAAGAGTCCCACGATACAA
*epe1Δ* - HR template	GTGAACTACTCAAGAATCATAAGCACGTGGGGATAAATATTCAATGGTAGCCGAAGGAAATAAAAAGTGCCGAGGTACTTCTTA AAAGTCCCAAAAATTA
*3xFLAG-epe1* - HR template	GAATATCAATGTCTTGATTTATAATGTCATCGTATTCAAGCCAGGAATCGCTGCCTCCTCCCTTGTCATCGTCATCCTTGTAGTCG ATGTCATGATCTTTATAATCACCGTCATGGTCTTTGTAGTCCATCTTAAAAGTCCCAAAAATTAATTGCTTACTAGCAAAAAGGTAA CTATAAA

Epe1 is a putative histone demethylase that acts to prevent heterochromatin island formation ( Sorida *et al.*, 2019; Wang *et al.*, 2015; Zofall *et al.*, 2012). Using *SpEDIT*, we deleted the *epe1 ^+^* coding sequence ( Figure 6C). The *epe1Δ* HR template employed contained 80 bp arms homologous to sequences immediately flanking the *epe1* ^+^ coding sequence as previously used for the deletion of other sequences ( Rodríguez-López *et al.*, 2016). Correct deletion of the *epe1* ^+^ coding sequence results in loss of the sgRNA target and PAM sequences. In addition, we seamlessly inserted a sequence to encode a 3xFLAG epitope tag between the *epe1* ^+^ gene promoter and the 5’ end of the *epe1* ^+^ coding sequence to allow the production of N-terminally 3xFLAG-tagged Epe1 without any associated selectable marker ( Figure 6D). To accomplish this, an in-frame *epe1-3xflag-epe1* HR template containing 50 bp arms homologous to the sequence immediately flanking the *epe1* ^+^ start codon was used. Correct insertion of the *epe1-3xflag-epe1 sequence* resulted in loss of both the sgRNA target and the PAM sequence. The resulting *epe1Δ* and 3xFLAG-Epe1 strains were recently utilised to study the role of Epe1 in ectopic heterochromatin island formation following caffeine exposure ( Torres-Garcia *et al.*, 2020). Notably, whole-genome sequencing of the *cup1:4xtetO* and *epe1Δ* strains revealed no additional genetic changes (SNPs or indels) in coding regions of the genome ( Torres-Garcia *et al.,* 2020).

In the four distinct genome editing scenarios described above, a maximum of eight primary transformants needed to be screened to obtain at least one that exhibited the desired sequence change. We therefore conclude that *SpEDIT* markedly speeds up the process of generating accurate insertion, deletion and tagging events at a variety of *S. pombe* loci.

## Discussion

Here we report the development of *SpEDIT*, an optimized CRISPR/Cas9 editing system and method for the fission yeast, *S. pombe* ( Figure 1 and Figure 2). *SpEDIT* makes use of Cas9 codon-optimised for expression in *S. pombe* that, coupled with the incorporation of a tDNA ^Ser^/HDV ribozyme sgRNA expression cassette ( Ryan *et al.*, 2014), achieves 100% efficiency in generating mutations at targeted *ade6 ^+^* or *ura4 ^+^* genes in asynchronous cells ( Figure 3). A high mutagenesis efficiency was also obtained with the pre-existing pMZ379 system in asynchronous cells ( Jacobs *et al.*, 2014; Rodríguez-López *et al.*, 2016). However, *SpEDIT* displayed reduced toxicity by removing the detrimental physiological effects associated with high humanised Cas9 endonuclease expression and consequently speeds up the genome editing process ( Figure 4). In addition, our analysis indicates that the use of G1-synchronized cell cultures for CRISPR/Cas9-mediated genome manipulation reduces the efficiency of targeted mutagenesis, with both the *SpEDIT* and pMZ379 systems, relative to asynchronous cultures ( Figure 3). G1 synchronization therefore represents an unnecessary time-consuming step in the genome editing process.

*SpEDIT* can be used to introduce simultaneous mutations at two non-homologous genes at distinct locations in the *S. pombe* genome ( Figure 5), and allows flexible engineering of seamless insertion, deletion and tagging events at *S. pombe* loci in the absence of linked selectable markers and without observed off-target sequence changes ( Figure 6). It is worth noting that many traditional *S. pombe* transformation protocols involve the use of carrier DNA. We advise against the use of carrier DNA as it has been shown to insert at many locations in resulting transformants, causing unplanned off-target mutations ( Longmuir *et al.*, 2019).

Besides achieving high mutagenesis efficiency, the greatest advance of *SpEDIT* is a very simple cloning protocol allowing sgRNA target sequences to be inserted with minimal effort into the Cas9-bearing pLSB plasmid through a one-step Golden Gate reaction. Candidate sgRNA-bearing clones are easily visualized by loss of the GFP placeholder, negating the need for repetitive screening of numerous *E. coli* colonies by laborious plasmid purification and inspection.

Recently it was reported that homology arms of as short as 25 bp flanking each side of a cleavage site can be used to successfully introduce point mutations and epitope tags at *S. pombe* loci ( Hayashi & Tanaka, 2019). Further analyses will be required to determine whether HR templates with such short homology arms are as efficient as longer arms when combined with *SpEDIT.* In addition, the tDNA/HDV ribozyme sgRNA expression cassette that was originally developed for *S. cerevisiae* has been used to express up to three tandem HDV-sgRNAs from a single tDNA RNAPIII promoter with 80% mutagenesis efficiency ( Ryan *et al.*, 2014). This suggests that a similar approach could be used with *SpEDIT* to simultaneously express multiple different sgRNAs that target a single locus or many distinct loci.

In summary, the combination of the CRISPR4P algorithm ( Rodríguez-López *et al.*, 2016), that conveniently aids the identification of suitable sgRNAs across the *S. pombe* genome, with *SpEDIT,* which provides a straightforward and user-friendly experimental method, markedly enhances the capabilities of CRISPR/Cas9-mediated genome editing in *S. pombe.* We anticipate *SpEDIT* will permit the broad application of genome editing procedures to fission yeast in order to explore diverse biological questions in this model fungal system.

## Methods

### pLSB construction

For pLSB-NAT construction, the Cas9 gene and the strong *adh1* promoter present in pMZ379 (plasmid generated by Mikel Zaratiegui and provided by Jürg Bähler ( Rodríguez-López *et al.*, 2016)), were replaced by a Cas9 gene codon-optimised for *S. pombe* (custom synthesised, Gen9) and the medium strength *adh15* promoter (from pRAD15, gift from Yoshi Watanabe), respectively, via Gibson Assembly (New England Biolabs, Cat# E2611). Next, the rrk1/HHR sgRNA cassette present in pMZ379 was replaced by the tDNA/HDV sgRNA cassette (custom synthesised, Gen9) via Gibson Assembly (New England Biolabs, Cat# E2611). *Bsa*I sites flanking the GFP placeholder in the tDNA/HDV sgRNA cassette were then introduced using Q5 Site-Directed Mutagenesis Kit (New England Biolabs, Cat# E0554).

To construct pLSB versions with *kanMX6* (pLSB-Kan, G418 resistance) or *hphMX6* (pLSB-Hyg, hygromycin resistance) selectable markers, the *natMX6* gene from pLSB-NAT was replaced by the *kanMX6 or hphMX6* genes from *pFA6a-kanMX6* ( Bähler *et al.*, 1998) *or pFA6a-hphMX6* ( Hentges *et al.*, 2005) plasmids, respectively, by Gibson Assembly (New England Biolabs, Cat# E2611).

sgRNA and HR template sequences can be found in Table 1. All sgRNA sequences used were obtained using CRISPR4P ( Rodríguez-López *et al.*, 2016). Oligonucleotide sequences are listed in Table 2.

**Table 2.** Oligonucleotides used in this study.

Name	Sequence
Making *ade6* – sgRNA - F	CtagaGGTCTCgGACTTTGATAGCAACAGTGGCGACGTTTcGAGACCcttCC
Making *ade6* – sgRNA - R	GGaagGGTCTCgAAACGTCGCCACTGTTGCTATCAAAGTCcGAGACCtctaG
Making *ura4* – sgRNA - F	CtagaGGTCTCgGACTCCTTGTATAATACCCTCGCCGTTTcGAGACCcttCC
Making *ura4* – sgRNA - R	GGaagGGTCTCgAAACGGCGAGGGTATTATACAAGGAGTCcGAGACCtctaG
Making *meu27* - sgRNA - F	CtagaGGTCTCgGACTTATTAGCCTTTGAAGGATTTGTTTcGAGACCcttCC
Making *meu27* - sgRNA - R	GGaagGGTCTCgAAACAAATCCTTCAAAGGCTAATAAGTCcGAGACCtctaG
Making *clr5* – sgRNA - F	CtagaGGTCTCgGACTAGCTTGTGGCTGACCGTTAAGTTTcGAGACCcttCC
Making *clr5* – sgRNA - R	GGaagGGTCTCgAAACTTAACGGTCAGCCACAAGCTAGTCcGAGACCtctaG
Making *cup1:4xtetO* – sgRNA - F	CtagaGGTCTCgGACTATTTCTTTTGCTTTACGGTCGTTTcGAGACCcttCC
Making *cup1:4xtetO* – sgRNA - R	GGaagGGTCTCgAAACGACCGTAAAGCAAAAGAAATAGTCcGAGACCtctaG
Making cup1-GFP – sgRNA - F	CTAGAGGTCTCGGACTGCTCAGGCTAAACGTCGGAAGTTTCGAGACCCTTCC
Making cup1-GFP – sgRNA - R	GGAAGGGTCTCGAAACTTCCGACGTTTAGCCTGAGCAGTCCGAGACCTCTAG
Making *epe1* – sgRNA - F	CtagaGGTCTCgGACTGGACTTTTAAGATGGATTCCGTTTcGAGACCcttCC
Making *epe1* – sgRNA - R	GGaagGGTCTCgAAACGGAATCCATCTTAAAAGTCCAGTCcGAGACCtctaG
Making *ade6* - HR template - F	GCAATGACACCTCTTCCAGTAATCGGCG TTCCTGTAAAAGGAAGCACTCTTGACGG AGTTGACTCTCTTTA GTCTATTGTTCAGA TGCCTCGAGGTGTCT
Making *ade6* - HR template - R	GGAGGGTTGAAATGTAGCAAGTATACGA CAGGCTAAAATACCGGCATTTTGGCTATT TATTGATAGCAACA GTGGCGACCTAGACA CCTCGAGGCATCTGA
Making *ura4* - HR template - F	TTGGAAGACATTTCAGCCAAAAGCAAGA GACCACGTCCCAAAGGTAAACCAACTTC TTTGAGGCCTTGTAT AATACCCTCGCCCT ACACTGTATGGCAAT
Making *ura4* - HR template - R	ATTCGCAGACATTGGAAATACCGTCAAG CTACAATATGCATCTGGTGTGTACAAAAT TTAGTCTTGGGCTCA TATCACAAATTGCC ATACAGTGTAGGGC
Making *meu27* - HR template - F	GCCAAAATCAATAGAGAACAATTATACTTTAAAAAAAAAAAATGAAGAAGGCTTCTTAAGTCAACAGGAAAAT AAGTATTCAAATCAAAATCCTTCAAAG
Making *meu27* - HR template - R	GAGGTGCCGCCCAATTGCAGTATACAAGCTATGAATGTTATTGGCTTGCTTACGCCGAGCTTGTCGAAAAGG TTATTAGCCTTTGAAGGATTTTGATTTG
Making *clr5* - HR template - F	CTTATTTGCAGCAGCCTTTCCAAATACCCTCTCAACGTTTCTCTCGACAGCAACAATCTCATCCATTCCCTGCT GCTCAACATGCAGTTAACGGTTAGCC
Making *clr5* - HR template - R	CAGATTGGTTTGAAGAAGCAAACATGGTGGAGCCCATTGGGACATTTCTAGATTGGTAGATGAAAGGATAC AAAGCTTGTGGCTAACCGTTAACTGCATG
Making *cup1:4xtetO* - HR template - 1 - F	TTGAATTAATTCATAGAGTATGATAAAAATTGATAGTAAATTCATTGG
Making *cup1:4xtetO* - HR template - 1 - R	cactagtaggccttgATGCATGCTAATAAATCATCGTAACTCAAGTAG
Making *cup1:4xtetO* - HR template - 2 - F	TTTATTAGCATGCATcaaggcctactagtgcatgca
Making *cup1:4xtetO* - HR template - 2 - R	TTTTTTTTTTCATAAATATTTActggatttcgtttacctcaccacc
Making *cup1:4xtetO* - HR template - 3 - F	tggtgaggtaaacgaaatccagTAAATATTTATGAAAAAAAAAATAAATGATTCATAACAAGCAGATGAAAA
Making *cup1:4xtetO* - HR template - 3 - R	TTTGTAATGTATAATCTTCATTTATTTTGAAGAGTCCTAATTCGT
Making cup1-GFP - HR template - F	ATGACGAATTAGGACTCTTCAAAATAAATGAAGATTATACATTACAAACTTTGGTCTGACTTTTTAAAGCACAC GATTTGCTATTTGTATAGTTCATCCA
Making cup1-GFP - HR template - R	TTGTATCGTGGGACTCTTTGTCAGACATTCAGCTCAGGCTAAACGTCGGAAAAGTTCTTAAAAAGTCAGTCA AAAAAAGGAGTAAAGGAGAAGAACTTTT
Making *epe1Δ* - HR template - F	GTGAACTACTCAAGAATCATAAGCACGTGGGGATAAATATTCAATGGTAGCCGAAGGAAATAAAAAGTGCCG AGGTACTTCTTAAAAGTCCCAAAAATTA
Making *epe1Δ* - HR template - R	CCATAGAATCTCCTTAGTTTGCATCGCAATTTTATAGTTACCTTTTTGCTAGTAAGCAATTAATTTTTGGGACTT TTAAGAAGTACCTCGGCACTTTTTA
Making *3xFLAG-epe1* - HR template - F	TTTATAGTTACCTTTTTGCTAGTAAGCAATTAATTTTTGGGACTTTTAAGATGGACTACAAAGACCATGACGGT GATTATAAAGATCATGACATCGACTA
Making *3xFLAG-epe1* - HR template - R	GAATATCAATGTCTTGATTTATAATGTCATCGTATTCAAGCCAGGAATCGCTGCCTCCTCCCTTGTCATCGTCAT CCTTGTAGTCGATGTCATGATCTTT
Checking mutations *ade6* - F	TTGTTTCAGCTCACCGCACA
Checking mutations *ade6* - R	AAAGCAAGCAAAATCATTTAACAGT
Checking mutations *ura4* - F	GCTCCATAGACTCCACGACC
Checking mutations *ura4* - R	TTGTCAGTCGCGGTCGATTT
Checking mutations *meu27* - F	AAATTTGCGCTCCTCTCTGC
Checking mutations *meu27* - R	GTTTGGTATTTACGAGCTGCCA
Checking mutations *clr5* - F	CACACAATGCGCACTCTTCT
Checking mutations *clr5* - R	ACAGCAGTTGGTCCGTTAGA
Checking *cup1:4xtetO* – F (A1)	GGTTAGGCAGAAGACTTGAGCA
Checking *cup1:4xtetO* – R (A2)	ATCATCACTTGCATTCACTTCTCT
Checking cup1-GFP - F	GGCGAAGCTTTTAAGTCTGAAGG
Checking cup1-GFP - R	GCTGTCCCACTCTTACCACA
Checking *epe1Δ* – F (C1)	CAAATCTAACGAGTTTGCCTGC
Checking *epe1Δ* – R (C3)	GCAAACAACGAGTCAAAGTGGA
Checking *epe1 cds* – F (C2)	GGGCGAGCGGACAATCATAA
Checking *3xFLAG-epe1* - F	AGTGAGGCTGTGCAAAGGAA
Checking *3xFLAG-epe1* - R	TCTAACGAGTTTGCCTGCTT
M13F	GTAAAACGACGGCCAGT

### Yeast strains and manipulations

Standard methods were used for fission yeast growth, genetics and manipulation ( Moreno *et al.*, 1991). *S. pombe* strains generated in this study are described in Table 3. 972 *h ^-^* wild-type cells were used for all experiments.

**Table 3.** *Schizosaccharomyces pombe* strains used in this study.

Strain number	Name	Description
143	wt	*h-* ED972 wild-type
B4752	Clr5-Q264STOP Meu27-S100Y	*h- clr5-Q264STOP meu27-S100Y*
B3808	*cup1:4xtetO*	*h- 4xtetO 3' of cup1 leu1-32 (cup1=SPBC17G9.13c)*
B4567	Cup1-GFP	*h- cup1-GFP (cup1=SPBC17G9.13c)*
B4621	*epe1Δ*	*h- epe1Δ*
B4958	3xFLAG-Epe1	*h- 3xFLAG-epe1*

Competent cryopreserved G1-synchronized *S. pombe* cells were prepared as described in Rodríguez-López *et al.* (2016). Wild-type cells were grown to a concentration of 1×10 ^7^ cells per mL in EMM media and harvested at 3500 x g for 2 min. Cells were then resuspended in EMM-N and incubated for 2 hrs at 25°C until cells were of a small round morphology. Following three washes with ice-cold H _2_O, cells were resuspended in 2 mL of ice-cold 30% glycerol, 0.1 M lithium acetate (pH 4.9) and 50 μL aliquots were made and stored at -80°C until further usage.

Competent cryopreserved G1-synchronized *S. pombe* cells were transformed using the lithium acetate/PEG method ( Sabatinos & Forsburg, 2010), as described in Rodríguez-López *et al.*, 2016. Aliquots of cryopreserved, G1-synchronised cells were thawed at 40°C for 2 min. 200 ng of empty or sgRNA-containing pLSB or pMZ379 plasmid DNA, 250–1000 ng of HR template DNA (when applicable) and 145 μL of 50% PEG 4000 were added to the cells and mixed thoroughly. Note that herring sperm DNA was omitted due to concerns regarding the erroneous integration of these fragments at the targeted loci as described in Longmuir *et al.* (2019). Cells were then resuspended in 1 mL of EMM-N and incubated for 16 hrs at room temperature. Following incubation, cells were resuspended in 500 μL H _2_O and plated onto YES plus cloNAT media. Plates were incubated at 32°C for 3–5 days.

Asynchronous cultures of *S. pombe* cells were transformed by electroporation. 50 mL cultures were grown to log phase (5×10 ^6^ to 1×10 ^7^ cells per mL) in YES media and harvested at 3500 x g for 2 min. Cells were resuspended in 5 mL of pre-transformation buffer (25 mM DTT, 0.6 M sorbitol, 20 mM HEPES, pH 7.6) and incubated at 32°C for 10 min. Cells were then washed three times in 10 mL of 1.2 M ice-cold sorbitol and resuspended in 500 μL of 1.2 M ice-cold sorbitol. 200 μL cells were added to 200 ng of empty or sgRNA-loaded pLSB or pMZ379 plasmid DNA and 250–1000 ng of HR template DNA (when applicable) in an ice-cold electroporation cuvette. Cells were pulsed using a Gene Pulser II electroporation system (Bio-Rad) with *S. pombe* settings: 2.25 kV, 200 Ω and 25 μF. Immediately following pulse, cells were rapidly mixed with 500 μL of 1.2 M ice-cold sorbitol. Cells were grown overnight in 10 mL of non-selective YES medium before plating on selection (YES plus cloNAT, YES plus G418 or YES plus hygromycin depending on pLSB version used). Plates were incubated at 32°C for 3-5 days. cloNAT, nourseothricin.

To assess colony area of pLSB- or pMZ379-harbouring cloNAT-resistant colonies, plates were scanned after four days of incubation. Images were then analysed using ImageJ (v1.51) (analyse particles) with default settings.

### Assessing mutations at the *ade6* and *ura4* loci

Colonies harbouring mutations at the target genes *ade6 ^+^* or *ura4 ^+^* were identified through a replica-plating assay. cloNAT-resistant colonies were individually picked from YES plus cloNAT plates, re-streaked onto YES plates without selection and incubated at 32°C for two days. Isolates were then replica-plated onto the following plates: YES, YES 1/10 adenine (to examine *ade6 ^+^* mutations), PMG minus uracil and PMG plus 5-fluoroorotic acid (to examine *ura4 ^+^* mutations). Plates were incubated at 32°C for 2–4 days and then visually examined.

### Colony PCR and Sanger sequencing

A small amount of fission yeast cells (~1 × 10 ^4^) from individual single colonies was incubated in 10 μL of SPZ buffer (1.2 M sorbitol, 100 mM sodium phosphate, 2.5 mg/mL Zymolyase 100T (AMS Biotechnology)) at 37°C for approximately 1 hr. 40 μL H _2_O was added to the cells and 5 μL of the extract was used as a PCR template. PCRs were performed using 0.5U *Taq* Polymerase (Roche, Cat# 11147633103), 1 μM oligonucleotides, and the following conditions on a standard thermocycler: 94°C for 4 min; 29 cycles of 94°C for 30 sec, 55°C for 30 sec and 72°C for 1 min; and a final step of 72°C for 5 min. PCR products were analysed on a 1% agarose gel, purified using Monarch PCR Cleanup Kit (New England Biolabs, Cat# T1030) and sequenced using BigDye Terminator Cycle sequencing kit (Thermo Scientific, Cat# 4337458).

### Protein extraction and western analysis

Protein samples were prepared as previously described ( Torres-Garcia *et al.*, 2020). Western blotting detection was performed using mouse monoclonal anti-FLAG-HRP (Sigma, Cat# A8591, RRID:AB_439702), rabbit polyclonal anti-GFP (Invitrogen, Cat# A11122, RRID:AB_2307355) and goat polyclonal anti-rabbit-HRP (Sigma, Cat# A6154, RRID:AB_258284).

### *SpEDIT* protocol

A convenient protocol card of this procedure can be found by visiting *allshirelab.com/spedit* or by scanning the QR code in Figure 1.

***Before you begin***

●   Download required DNA sequences at *allshirelab.com/spedit* , on Zenodo ( Torres-Garcia, 2020) or by scanning the QR code in Figure 1

***Required reagents***

●   pLSB vector (75 ng/μL) – Available on request

●   NEB Golden Gate Assembly Kit ( *Bsa*I-HF v2) – NEB #E1601S

●   sgRNA fragment for Golden Gate assembly (1 ng/μL) – See below for design and preparation

●   HR template (250-1000 ng) – See below for design and preparation

***sgRNA design***

●   Find a suitable sgRNA targeting the gene of interest using CRISPR4P ( Rodríguez-López *et al.*, 2016)

●   Copy the 20 bp sgRNA sequence in place of ‘your 20 bp targeting RNA’ in ‘GG_sgRNA_template.dna’ file

●   Order 52-nt forward and reverse sgRNA oligonucleotides as indicated in the ‘GG_sgRNA_template.dna’ file

***sgRNA fragment preparation***

●   Anneal sgRNA oligonucleotides

○ Mix 5 μL of 100 μM forward and reverse sgRNA oligonucleotides
○ Heat to 95°C for 3 min and cool down to room temperature slowly (e.g. -1°C/30 sec)
○ Add 1 μL of annealed mix to 1.5 mL of H _2_O. This dilution corresponds to approximately 1 ng/μL annealed sgRNA fragment.

***Golden Gate reaction***

●   Mix the following components in a PCR tube:

○ pLSB plasmid (75 ng/μL) – 0.5 μL
○ Annealed sgRNA fragment (1 ng/μL) – 0.5 μL
○ T4 DNA Ligase Buffer (10x) – 1 μL
○ NEB Golden Gate Assembly Mix ( *Bsa*I-HF v2) – 0.5 μL
○ H _2_O – 7.5 μL

●   Incubate at 37°C for 1 hr

●   Incubate at 60°C for 5 min

●   Transform into *Escherichia coli* by heat shock:

○ 1 μL of Golden Gate reaction to 25 μL of *DH5-alpha* or *10-Beta E. coli* cells
○ Place the mixture on ice for 30 min
○ Heat shock at exactly 42°C for exactly 30 sec
○ Place on ice for 5 min
○ Add 475 μL SOC media and recover cells at 37°C for 1 hr
○ Plate 200 μL / 100 μL / 50 μL / rest on LB plus ampicillin

●   Select four *E. coli* colonies and set up liquid cultures in LB plus ampicillin

●   Isolate plasmid (miniprep)

○ IMPORTANT: do not miniprep culture if green – these are GFP-containing clones where the Golden Gate reaction did not occur
○ One miniprep (approximately 200 ng/μL) should be sufficient for many *S. pombe* transformations (200 ng/transformation)

***Plasmid check***

●   Digest plasmid using *Nco*I

○ This digest allows sgRNA-containing plasmids to be distinguished from those containing GFP

●   Sequence sgRNA-containing plasmids using M13F oligonucleotide (see Table 2) to confirm sgRNA insertion

***HR template design and preparation***

●   The HR template should contain:

○ Your desired mutation: point mutation, insertion, deletion or tag

○ At least 80 bp homology on each side relative to the cleavage site (3 bp upstream of PAM sequence (NGG))

○ A mutation that disrupts the PAM sequence to avoid repeated DSBs
  ▪ If the total size of the HR template is equal or smaller than 180 bp, we recommend generating HR templates by PCR using oligonucleotides with overlapping at their 3’ end as described in Rodríguez-López *et al.*, 2016
  ▪ If the total size of the HR template is larger than 180 bp, we recommend using a fusion PCR construct containing homology arms to the target site (see HR template for *cup1:4xtetO* in Table 1)

***S. pombe transformation***

●   Transform *S. pombe* cells using your preferred method with sgRNA-loaded pLSB plasmid (200 ng) and HR template (500-1000 ng)

●   Grow non-selectively o/n on YES plates or liquid

●   Plate on YES plus cloNAT (or YES plus G418 or YES plus hygromycin depending on pLSB version used)

●   Re-streak transformant colonies to non-selective media to allow loss of plasmid

●   Amplify region of interest by colony PCR and sequence amplicon to confirm mutation

## Data availability

### Underlying data

Whole-genome sequencing data for relevant strains are available on GEO, Accession number GSE138436: https://identifiers.org/geo:GSE138436 ( Torres-Garcia *et al.,* 2020).

Zenodo: *SpEDIT*: A fast and efficient CRISPR/Cas9 method for fission yeast.

https://doi.org/10.5281/zenodo.4247568 ( Torres-Garcia, 2020)

This project contains the following underlying data:

- Fig_3C.csv (Raw mutagenesis efficiency data underlying Figure 3C)
- Fig_3D.csv (Raw mutagenesis efficiency data underlying Figure 3D)
- Fig_4A.csv (Raw colony area measurements underlying Figure 4A)
- Fig_4B.csv (Raw colony area measurements underlying Figure 4B)
- F6_uncropped.pdf (Original, uncropped PCR gel and western blot images from Figure 6)

### Extended data

Zenodo: *SpEDIT*: A fast and efficient CRISPR/Cas9 method for fission yeast.

https://doi.org/10.5281/zenodo.4247568 ( Torres-Garcia, 2020)

This project contains the following extended data:

- GG_sgRNA_template.dna (sgRNA sequence)
- pLSB_Allshire.dna (pLSB plasmid sequence)

Data are available under the terms of the Creative Commons Zero "No rights reserved" data waiver (CC0 1.0 Public domain dedication).
