# Supplementary material for: SpEDIT: A fast and efficient CRISPR/Cas9 method for fission yeast
Source: Wellcome Open Res. 2020 Nov 24;5:274. [Version 1] doi: 10.12688/wellcomeopenres.16405.1 (PMC7721064; doi:10.12688/wellcomeopenres.16405.1)
Supplement: Peer review history

# Peer review: Reviewer response for version 1

**DOI:** 10.21956/wellcomeopenres.18048.r41572

**Published:** 2021-01-15

**Authors:** Sigurd Braun, Thomas van Emden

**Affiliations:** 1 Ludwig Maximilians University of Munich, Martinsried, Germany; 2 Ludwig Maxilians University of Munich, Martinsried, Germany

Torres-Garcia and colleagues report an improved method for scarless genome editing in *S. pombe* by CRISPR/Cas9. This method coined *SpEDIT* overcomes limitations of previous CRISPR protocols (e.g., Jacobs *et al*., 2014; Rodriguez-Lopez *et al*., 2016) ^1^^,^^2^ that are plagued by tedious cloning procedures to integrate sgRNAs into suitable expression vectors and the toxicity of overexpressed Cas9 endonuclease.

 For their new expression system, the authors replaced the Cas9 gene with an *S. pombe* codon-optimized Cas9 gene expressed from the medium-strength *adh15* promoter, a derivative of the strong *adh1* promoter (Yamagishi *et al*. 2008) ^3^. For the expression of the sgRNA, they adopted an expression system successfully used in *S. cerevisiae* (Ryan *et al*. 2014) ^4^, consisting of an RNA polymerase III-driven tRNA gene/hepatitis delta virus ribozyme (HDV) sequence that creates a defined 5’ transcript and improves sgRNA stability and genome editing. The tDNA/HDV cassette further allowed insertion of two *BsaI* restriction sites for one-step cloning of the sgRNA by the Golding Gate cloning system. In addition, they inserted a GFP sequence as a placeholder within the *BsaI *sites for visual inspection of positive *E. coli* clones. Together, this modular design significantly improved the cloning procedure compared to previous methods that are hampered by the use of an inconvenient restriction enzyme ( *CspCI) *or tedious PCR-based cloning methods. Moreover, by offering multiple expression vectors with different selection markers ( *natMX6*, *kanMX, *or *hphMX*), *SpEDIT* allows versatile use in different strain backgrounds.

 The authors demonstrate that *SpEDIT *works 100% efficiently when introducing single-point mutations into the *ura4+* and *ade6+* gene nearby PAM sites. They further showed that using G1-synchronized cells for transformation is not necessary to achieve high efficiency, as was previously reported (Rodriguez-Lopez *et al*., 2016) ^2^. Moreover, they demonstrated that *SpEDIT *can also be applied for other gene-editing steps, like gene deletions, insertions, or truncations, as well as the simultaneous editing of two unrelated genetic loci using expression plasmids with different selection markers. However, the efficiency of these more challenging genetic alteration was significantly lower (about 10-20%) compared to introducing single point mutations.

**Conclusion:**

 The CRISPR/Cas9 system presented here overcomes previous technical hurdles and significantly improves the usability of Cas9 based genome editing in *S. pombe. *The manuscript is well written, and the protocol is easy to follow. The authors have shared their system with my lab, and we have successfully used it to introduce mutations into several genes, confirming the efficiency of this system. Though, we did notice that editing at some loci is more challenging. Previous studies (e.g., Rodriguez-Lopez *et al*., 2016) ^2^ have analyzed a larger number of genomic targets and compared different sgRNAs to assess the efficiency of the editing method. Thus, the reported high efficiency for *ade6+* and *ura4+* may not always be achieved for other genomic targets. Furthermore, while several parameters have been changed compared to existing methods, it is sometimes unclear how these changes (e.g., codon-optimization, reduced expression) have improved efficiency (see comments below). Maybe, the authors could provide more information in a revised version, which may clarify some of the points mentioned below.

**Comments and questions:**

- For comparing SpEDIT with a previous method reported by the Bähler Lab (Rodriguez-Lopez *et al*., 2016) ^2^, the authors assessed mutagenesis efficiency and correct editing in Figure 3C, D. When using matching sgRNAs and HR templates, both methods seem to work equally efficiently for asynchronous cells. In contrast, differences were found for mismatching or no templates, as demonstrated by plotting mutant phenotypes as percentage relative to the total number tested. However, we wonder whether plotting the actual number of clones with mutant phenotypes would be more informative than plotting percentage. While percentage includes information on false positives, which is in principle meaningful, this analysis may be skewed when analyzing different total numbers of samples, especially for low numbers. For instance, for analyzing ade6 mutations, both pLSB with pMZ379 resulted in similar numbers of clones with mutant phenotypes when using the correct HR template (150 vs. 150), non-correct HR template (12 vs. 26), or no HR template (0 vs. 1). However, by plotting percentages (i.e., number of clones with mutant phenotypes relative to total) the difference between both plasmids seems much larger (e.g., 0% vs. 100% for no template). This may reflect noise due to low sample numbers, but this does not immediately become clear when looking at the graphs. The same applies to the tables shown in Figure 3E. Also, providing here exact numbers (e.g., 5/5 or 4/5) seems more accurate and informative (especially when only 1 or 2 clones have been tested).
- The authors emphasize that they codon-optimize the Cas9 sequence and that its reduced expression results in decreased toxicity; however, how does this contribute to improved editing or a more user-friendly procedure? While the pLSB plasmid decreased toxicity in asynchronous cells compared to pMZ379, this did not improve gene editing efficiency for inserting single point mutations in *ura4+* and *ade6+*. Did the authors also compare the two plasmids for editing other targets or introducing more rate-limiting changes (e.g., deletions or insertions)?
- What is the expression strength of *adh15* promoter? (for comparison: for the *adh1* gene, 400 RNA molecules/cell have been reported).
- How does using electroporation affects transformation efficiency compared to chemical transformation? Is there a difference in the number of colonies after transformation with pLSB vs. pMZ379? Along the same line, to what extent is the comparison of gene editing using asynchronous and G1 synchronized cells confounded by using different transformation methods?

Is the rationale for developing the new method (or application) clearly explained?

Yes

Is the description of the method technically sound?

Yes

Are the conclusions about the method and its performance adequately supported by the findings presented in the article?

Yes

If any results are presented, are all the source data underlying the results available to ensure full reproducibility?

Yes

Are sufficient details provided to allow replication of the method development and its use by others?

Yes

Reviewer Expertise:

Fission yeast, genetics, molecular biology.

We confirm that we have read this submission and believe that we have an appropriate level of expertise to confirm that it is of an acceptable scientific standard.

## References

[1] : Implementation of the CRISPR-Cas9 system in fission yeast. *Nat Commun*.2014;5: 10.1038/ncomms6344 5344 10.1038/ncomms6344 25352017PMC4215166

[2] : A CRISPR/Cas9-based method and primer design tool for seamless genome editing in fission yeast. *Wellcome Open Research*.2016;1: 10.12688/wellcomeopenres.10038.1 10.12688/wellcomeopenres.10038.1 28612052PMC5445975

[3] : Heterochromatin links to centromeric protection by recruiting shugoshin. *Nature*.2008;455(7210) : 10.1038/nature07217 251-5 10.1038/nature07217 18716626

[4] : Selection of chromosomal DNA libraries using a multiplex CRISPR system. *Elife*.2014;3: 10.7554/eLife.03703 10.7554/eLife.03703 25139909PMC4161972

# Peer review: Reviewer response for version 1

**DOI:** 10.21956/wellcomeopenres.18048.r41573

**Published:** 2020-12-04

**Authors:** Sophie Martin

**Affiliations:** 1 Universite de Lausanne, Lausanne, Switzerland

This manuscript by Torres-Garcia and colleagues presents an improved CRISPR/Cas9 method for genome engineering in the fission yeast *S. pombe*. The main improvements over previously described methods are reduced toxicity of the Cas9 plasmid and greater ease of cloning of the sgRNA. They show that the method is highly efficient for introduction of point mutations and can also be used to introduce point mutations in two loci simultaneously or to insert or delete longer sequences.

 The manuscript is well presented and convincing. The authors have already distributed their reagents, and my lab has used them with success. I can thus confirm that the method is functional and easy to use. It is likely to become widely used in the community. My comments below are mainly asking for some clarification.

 One question that is not addressed is why Cas9 is toxic. The readout is small colony size, but whether this is linked to Cas9 binding the genome and/or inducing cuts is not clear. The authors cite a master thesis, which reports that toxicity is not linked to Cas9 catalytic activity, which is interesting, but to my knowledge this has not been probed further. I would suggest spelling out that the reason for toxicity is unclear and incite researchers to use the method with care.

 I was also puzzled by why the target site appears to be more efficiently mutated when cells are co-transformed with an irrelevant HR template than with no HR template (Figures 3C-D). Any thought about this?

 What is the efficiency of sequence insertion/tagging/deletion? The text states that 8 clones needed to be analysed to find at least 1 positive one. This suggests lower success rate than for the point mutations. It would be informative to state the results fully and make this point clear.

**Regarding the methodology:**

- The authors used Golden Gate to clone the sgRNA sequence in pLSB, but in principle other cloning strategies (including standard T4-ligase-based strategies) are possible. This would be good to indicate.
- A similar point goes for the transformation strategy. The authors state in the text that they used electroporation, which is not the most widely used strategy in yeast labs. However, in the protocol, they state that any transformation strategy can be used. Does this matter?
- In Figure 2C, I would suggest modifying the scheme to better indicate the difference of colour of the colonies (rather than cultures). The scheme seems to indicate that all will be picked and grown, and that the green cultures will be discarded, but in reality, one only picks from the non-green ones.

**Regarding data accessibility:**

- The whole-genome sequencing, stated to have revealed no additional genetic change, is not very easy to find in the cited manuscript, or even the GEO accession number provided. I would suggest a direct reference to sequencing of the strain. For instance, I could identify that one was accession GSM4407513, but was not sure of the others.
- In the data source for Figures 4A-B, the name of the image file that was likely quantified is not particularly useful without the actual image.

Finally, I would encourage the authors to deposit the pLSB vector in repository centers such as the Japanese National BioResource Project or Addgene.

Is the rationale for developing the new method (or application) clearly explained?

Yes

Is the description of the method technically sound?

Yes

Are the conclusions about the method and its performance adequately supported by the findings presented in the article?

Yes

If any results are presented, are all the source data underlying the results available to ensure full reproducibility?

Yes

Are sufficient details provided to allow replication of the method development and its use by others?

Yes

Reviewer Expertise:

Fission yeast, genetics, cell biology

I confirm that I have read this submission and believe that I have an appropriate level of expertise to confirm that it is of an acceptable scientific standard.

# Peer review: Reviewer response for version 1

**DOI:** 10.21956/wellcomeopenres.18048.r41575

**Published:** 2020-11-25

**Authors:** Antony Carr

**Affiliations:** 1 Genome Damage and Stability Centre, University of Sussex, Brighton, Sussex, BN1 9RQ, UK

This paper describes a useful refinement of CRISPR/Cas9 editing in fission yeast. It will be beneficial to the fission yeast community. The refinements include reduced expression of Cas9 and a better way to clone the sgRNA sequence. A range of applications are tested and a clear protocol provided.

 Minor points.

 Some readers do not know what Golden Gate cloning entails. A brief summary and an additional panel, for example in Figure 2, would be very helpful for those people.

 Abstract: "...driven by RNA polymerase III from a tRNA gene sequence." Feels clumsy.

 Page 3 second para: "...where strand end resection..." delete strand, or specify more detail.

 Page 4 second paragraph: "Insertion of sgRNA sequences targeting genomic regions of interest into the rrk1 sgRNA expression cassette in current CRISPR/Cas9 systems for *S. pombe* relies on slow and arduous cloning procedures involving either traditional restriction digestion (Hayashi & Tanaka, 2019; Jacobs *et al.*, 2014) or PCR over a long template (Rodríguez-López *et al*., 2016)." is a difficult sentence to read. It could be clarified and/or broken into several sentences.

 Page 4 third para: "The tDNA ^Ser^ acts as a RNAPIII promoter and the self-cleaving ribozyme protects and defines the 5’ end of the resulting sgRNA (Figure 2A and B)" I was expecting the figure to help explain how the ribozyme protects and defines the 5' end.

 Page 5 first para: "...pink colonies develop on low (1/10) adenine-containing plates..." Please state here or in the methods what the concentration is.

Is the rationale for developing the new method (or application) clearly explained?

Yes

Is the description of the method technically sound?

Yes

Are the conclusions about the method and its performance adequately supported by the findings presented in the article?

Yes

If any results are presented, are all the source data underlying the results available to ensure full reproducibility?

Yes

Are sufficient details provided to allow replication of the method development and its use by others?

Yes

Reviewer Expertise:

Fission yeast molecular genetics.

I confirm that I have read this submission and believe that I have an appropriate level of expertise to confirm that it is of an acceptable scientific standard.
